# Post-discharge experiences of Malaria patients in a Vhembe district village, South Africa

**DOI:** 10.4102/hsag.v30i0.3032

**Published:** 2025-08-19

**Authors:** Wavhudi Kwinda, Takalani R. Luhalima, Aluwani D. Mudzweda

**Affiliations:** 1 Department of Advanced Nursing Science, Faculty of Health Sciences, University of Venda, Thohoyandou, South Africa

**Keywords:** diagnosed, experiences, malaria, people, post-discharge, Vhembe District

## Abstract

**Background:**

People in South Africa have been diagnosed with malaria, including those coming from other countries. Malaria is still life-threatening, and people are still being diagnosed even after preventive measures have been developed.

**Aim:**

The study’s purpose was to determine the post-discharge experiences of people diagnosed with malaria at Mhinga village of Vhembe district, Limpopo province.

**Setting:**

The setting was at the participants’ homes at Mhinga village, Collins Chabane Municipality, Vhembe district, Limpopo.

**Methods:**

An exploratory, descriptive and contextual qualitative method was employed to attain a comprehensive understanding of the phenomenon. Audio recordings of semi-structured interviews focused on the post-discharge experiences of malaria patients in Mhinga village, Vhembe district, Limpopo, with participants selected through purposive sampling. Qualitative theme analysis was performed utilising codes, while adhering to trustworthiness and ethical considerations.

**Results:**

Two themes emerged: post-discharge experiences from Mhinga clinic and post-discharge experiences from the hospital. The findings described and expressed the post-discharge experiences of people diagnosed with malaria at Mhinga village, Vhembe district, which included complicated symptoms experienced after discharge from the Mhinga clinic and the transfer to Malamulele hospital.

**Conclusion:**

The study has concluded that the participants had experienced complicated symptoms of malaria even after obtaining treatment from the clinic and hospital.

**Contribution:**

This study highlights the potential reduction of admissions to the Limpopo Province Department of Health by increasing awareness of malaria prevention methods. It suggests strategies to combat malaria, such as enhancing surveillance systems, implementing vector control measures, conducting public awareness campaigns, equipping healthcare facilities, reducing mosquito breeding sites and collaborating with neighbouring regions for cross-border efforts.

## Introduction

Children under the age of five, women and girls, indigenous peoples, migrants, individuals with disabilities and those living in rural regions without access to healthcare are among the groups most at risk of contracting malaria, according to the World Health Organization (WHO 2024). A large portion of the population in the WHO African Region, which is responsible for around 95% of the fatalities, does not have access to the necessary resources to avoid, identify and cure the disease (WHO 2024). According to Ayanlade et al. (2022), malaria is still a big problem in South Africa and many other parts of the world. Makhado et al. (2022) found that malaria is still a problem in the Vhembe district of Limpopo province. Thus, it is vital to effectively assist patients in their recovery and prevent reoccurrence.

### Problem statement

Malaria remains a significant public health concern in the Vhembe district of Limpopo province, South Africa. Despite advancements in treatment and prevention, the post-discharge experiences of individuals diagnosed with malaria in this region are not well documented. This study aimed to explore the post-discharge experiences of people diagnosed with malaria in a selected village of Vhembe district, focusing on their health outcomes, adherence to follow-up care and the socio-economic and psychological challenges they face. Understanding the experiences is crucial for developing targeted interventions to improve post-discharge care and support for malaria patients in this community.

### Rationale of the study

Tshivhase, Mashau and Munyai (2022) conducted a study on selected villages about caregiver’s knowledge on malaria in children below the age of 12 years, and the study indicated that Mopani and Vhembe district had the most people who experienced malaria. Patrick et al. (2023) investigated how household living conditions and individual behaviours are associated with malaria risk in Limpopo. It was found that housing conditions such as overcrowding pressures and poor living environments were factors of malaria exposure. There has been a gap in the post-discharge experiences of people diagnosed with malaria in the villages of Vhembe district, and malaria has been causing death. If people are not knowledgeable about their experiences of malaria, it may lead to increased rates of morbidity and mortality and make it difficult to implement preventative measures. The proposed study objective was to explore and describe the post-discharge experiences of people diagnosed with malaria in Mhinga village of Vhembe district.

### Significance of the study

The findings of the study were necessary as it may benefit the community members, considering that malaria is one of the leading causes of death, and may give them knowledge about precautions and prevention methods against the spread of malaria. This study was of importance as people became more knowledgeable about malaria prevention methods and may reduce its spread. Unique contributions include relieving the Department of Health from possible reduction of admissions. Moreover, morbidity, mortality rate and costs for treating malaria might decrease. National guidelines concerning malaria such as mosquito avoidance and the use of antimalarial agents have been present and effective in preventing malaria; therefore, policymakers might also encourage the community to continue implementing preventative measures to reduce malaria cases.

### Theoretical framework

The health belief model (HBM) is defined as a psychological theoretical framework intended to guide the promotion of health-related behaviours and elimination of disease (Wong et al. 2021). The researcher applied the HBM to the study using the concepts of perceived susceptibility, perceived severity, perceived benefits, perceived barriers, cues to action and self-efficacy. A high number of people residing in Mhinga village have experienced and continue to experience malaria; therefore, malaria has been considered as a perceived threat to the participants of Mhinga village. The participants perceived the high risk of being infected with malaria and thus engaged in behaviours to reduce the chances of being infected. The findings of the study revealed that participants would wear long-sleeved clothing, sleep under bed nets, close windows before dark and use mosquito coils in order to reduce their chances of being infected. When the participants of Mhinga village perceived malaria as a significant threat, they engaged in actions to reduce its seriousness. The perceived severity in this study refers to the malaria manifestations and complications experienced. The findings of the study revealed that the manifestations experienced by the participants included diarrhoea, fever, abdominal pain, body pain, body weakness, constant headaches, nausea and vomiting. The results also showed that participants who experienced aphasia, inability to walk and extreme fatigue were diagnosed with complicated malaria and thus were more likely to take action to reduce the chances of being infected again. The advantages of taking preventative actions included the reduction of malaria infection, thus bringing freedom from the disease and saving lives, as malaria has been known to cause death. The findings of this study revealed that the Mhinga participants recognized the benefits of taking preventative actions, such as cleaning their yards. This was observed by the researcher during interviews, where participants expressed that they did not want to experience malaria again. Hence, they followed the preventative measures willingly, which outweighed the barriers. The Mhinga participants perceived certain barriers when they delayed malaria treatment. The findings of the study revealed that perceived barriers to seeking early treatment included a lack of knowledge of the infection, belief to heal naturally, childcare and employment needs. In this study, the findings revealed that participants decided to visit the clinic because of advice from parents and wives, the sickness of their children with the same symptoms and the previous illness of a family member who had experienced similar symptoms. The findings of the study revealed that most participants are taking preventative actions against malaria even from observation at their homes during interviews while maintaining clean yards and surroundings.

### Aim

The research purpose was to determine the post-discharge experiences of people diagnosed with malaria at a selected village of Vhembe district, Limpopo province, South Africa.

## Research methods and design

An exploratory, descriptive and contextual qualitative research approach was employed to gain a comprehensive understanding of the phenomenon (Bhandari 2020; Hassan 2024). The HBM guides the data collection and the development of the interview instrument for individuals recovering from malaria after hospital discharge in a village setting. The model’s core constructs – including perceived susceptibility, severity, benefits, barriers, cues to action and self-efficacy – were utilised to assess patients’ beliefs and attitudes towards malaria and its treatment. This provides valuable insights for improving post-discharge care and intervention strategies. This method allowed the researcher to investigate and articulate the post-discharge experiences of individuals diagnosed with malaria in Mhinga village. It facilitated the researcher’s comprehension of the experiences of individuals diagnosed with malaria in Mhinga village, Vhembe district, and enabled the researcher to gather responses from a diverse array of participants, thereby allowing them to express themselves authentically and enhancing the accuracy of the research.

### Setting

The investigation was conducted in Mhinga village, Collins Chabane Municipality, Vhembe district, Limpopo province, South Africa, irrespective of the education level, income, religion or marital status of the community members. The researcher chose the participants by visiting the Mhinga clinic to identify individuals who had been diagnosed with malaria, obtained their consent and conducted the research in their homes to ensure that the participants felt comfortable and at ease while answering interview questions. The researcher noticed that some of the homes were situated near streams or rivers and therefore were likely to be the most susceptible to malaria. Subsequently, some of the homes were located at greater distances from water bodies. The Kruger National Park is situated close to Mhinga village, where mosquitoes are more prevalent in forested regions.

### Sampling of institutions

Pryszlak (2024) stated that area sampling is a way to get information about a setting, environment or surroundings by collecting samples from the exact location where it appears to be taking place. The sampling area was at Mhinga village because it was the area under investigation as a hotspot of people who had been diagnosed and experienced malaria.

### Sampling of participants

Participants were selected by visiting Mhinga clinic to obtain a list of diagnosed individuals, along with their contact information and addresses. Authorisation to access the statistics was secured from the province, the district and subsequently the local clinic manager. The diagnosed individuals were contacted to verify their location. Being interviewed at their homes might make participants emotionally comfortable to answer the interview questions.

### Data collection

A semi-structured face-to-face interview with interview guide questions was used from July to October 2024 to gather information. The researcher asked participants to go into more detail about their post-discharge experiences on being diagnosed with malaria. Other questions were made based on what the researcher thought or saw during the conversation, where they asked a lot of questions and got a lot of information. Semi-structured questions were created in English, and the interviews were conducted in English; where participants did not understand, the researcher explained in a simple way. Data were gathered during the day on the weekends, which included people who worked during the week. People who took part were told that they had the right to end the interview whenever they wanted to protect their right to self-determination. After getting consent from the people who took part, audio recordings were used. Every day, a language expert turned audio clips into written work in English. The researcher talked to each person for about 15 min to 30 min. After reaching saturation with 10 participants, the researcher chose to include 16 additional participants to capture more diverse perspectives and enhance data authenticity.

### Data analysis

According to Hassan (2024), the data management and analysis plan details how the researcher will analyse and interpret the data to uncover significance. The researcher listened to an audio recorder and transcribed the interview, which a language expert translated into English. The researcher reread each transcription to familiarise herself with the data and sort similar and dissimilar concepts. Brink, Walt and Rensburg’s (2018) instructions guided data analysis. Firstly, the researcher analysed the data ‘hands-on’ by obtaining a lot of text-based data and thinking about its meaning and relationships. The researcher read the transcript numerous times to comprehend what the participants stated. Secondly, the researcher employed codes – groups of letters, numbers, symbols and words or sentences – to sort the data. Participants’ words were used to classify data into themes and subthemes. Subthemes were built from five themes. Researchers interpreted the data based on diagnosed patients’ post-discharge experiences. Finally, an explanatory code was utilised to clarify the possible meanings of these codes, which changed as the researcher learned more about the data. To prevent data misuse, the researcher’s laptop was password-protected.

### Measures to ensure trustworthiness

The researcher’s credibility was ensured by the researcher’s presence in the field with the participants for a period of 2 weeks. This allowed the researcher to become familiar with the distinctive characteristics of the participants, which included their perspectives, experiences and perceptions. The researcher increased the trustworthiness of their findings by conducting semi-structured interviews, using an audio recorder and taking field notes. In order to guarantee the dependability of the findings, the recordings and paperwork were kept safe so that the research could be repeated with participants in an environment that was different from the original one. For the purpose of confirming interpretations, findings and proposals, conformability was assured by listening to audio recordings in great detail. Transferability was ensured by providing a full description, which included the location, the individuals who participated and the procedures that were utilised to gather data. This allowed other researchers to arrive at findings and conclusions that were comparable to those that were obtained. In this manner, other researchers would be able to determine whether or not the findings were applicable to different contexts or whether or not they were transferable. The researcher’s credibility was ensured by the researcher’s presence in the field with the participants for a period of 2 weeks. This allowed the researcher to become familiar with the distinctive characteristics of the participants, which included their perspectives, experiences and perceptions. The researcher increased the trustworthiness of their findings by conducting semi-structured interviews, using an audio recorder and taking field notes. In order to guarantee the dependability of the findings, the recordings and paperwork were kept safe so that the research could be repeated with participants in an environment that was different from the original one. For the purpose of confirming interpretations, findings and proposals, conformability was assured by listening to audio recordings in great detail. Transferability was ensured by providing a full description, which included the location, the individuals who participated and the procedures that were utilised to gather data. This allowed other researchers to arrive at findings and conclusions that were comparable to those that were obtained. In this manner, other researchers would be able to determine whether or not the findings were applicable to different contexts or whether or not they were transferable.

### Ethical considerations

In order to ensure quality assurance, the researcher presented the proposal to the University of Venda for evaluation, and the proposal was then forwarded to the Higher Degrees Ethics Committee for approval and quality assurance. The proposal was then forwarded to the Limpopo Department of Health for approval. Ethical approval was obtained from the University of Venda Higher Degrees Ethics Committee with clearance no. FHS/24/PDC/01/0404. The proposal was submitted to the Limpopo Department of Health for approval. The Vhembe District Department of Health issued a permission letter. The Chief of Mhinga village or relevant stakeholders in the community granted permission to conduct the study. Participants then gave a signed consent voluntarily, and ethical principles were followed during community engagement. Participants were assured that because participation was voluntary, they could withdraw from the study at any moment without being punished, protecting their right to self-determination. The researcher maintained confidentiality by employing codes instead of the participants’ actual names, which resulted in anonymity. This was done to ensure that the data could not be connected to the participants. All participants remained engaged till the conclusion without any withdrawals. Records were securely stored in a password-protected device, with a copy accessible exclusively to the researchers.

## Results

The demographic data of participants are presented in Table 1. A total of 26 participants were identified and interviewed about their post-discharge experiences when they got sick with malaria at Mhinga village of Vhembe district, Limpopo province. Both males and females were included, provided that they met the inclusion criteria. Emerging themes and sub-themes are shown in Table 2 below.

**TABLE 1:** Demographic summary.

Demographic variable	Category	Total number
Spoken language	Xitsonga	16
	Tshivenda	6
	English	4
**Total**		**26**
Number of episodes of malaria	One episode	19
	Two episodes	7
**Total**		**26**
Gender	Male	15
	Female	11
**Total**		**26**
Age group	20-29	4
	30-39	5
	40-49	12
	50-59	1
	60-69	2
	70-79	1
	80-89	1
**Total**		**26**
Year of experience of malaria	2024	3
	2023	14
	2022	7
	2021	2
**Total**		**26**

**TABLE 2:** Themes and sub-themes from the findings.

Emerged themes	Sub-themes	Categories
Theme 1: Post-discharge experience from Mhinga clinic	1.1. Immune system	Fever and chills Body pain
	1.2. Gastrointestinal system	Poor appetite Diarrhoea
	1.3. Central nervous system	Pins and needle sensation Drowsiness Aphasia Confusion
	1.4. Central and peripheral nervous system	Inability to sleep
	1.5. Musculoskeletal system	Body weakness
Theme 2: Post-discharge experience from the hospital	2.1 Post-discharge symptoms from the hospital	Mild body weakness
	2.3 Musculoskeletal system	

### Theme 1: Post-discharge experience from Mhinga clinic

Participants’ responses indicated that symptoms emerged at home after treatment at Mhinga clinic, some of which were similar to, and others different from, those experienced before treatment. Some symptoms got severe, forcing participants to drive straight to the hospital without stopping at the clinic as directed.

#### Post-discharge symptoms from the clinic

The study found that following clinic discharge, patients reported fever and chills, body pain, poor appetite, diarrhoea, pins and needle sensation, drowsiness, aphasia, inability to sleep and body weakness. These symptoms are detailed as follows.

Sub-theme 1.1: Immune system: The study’s findings revealed that fever, chills and body pains were continuously experienced even after discharge from the clinic. Participants expressed that the symptoms would disturb them from sleeping and contribute to feelings of not being well at all. Participants could not understand why the pain was still there and experienced it when they got back home. Participants indicated that they were recovering bit by bit from these symptoms until they faded off completely, as evidenced by the following participant’s quotation:

‘I was still feeling the cold and hotness during the four days of drinking pills but became fine after.’ (Participant 23, female, aged 25)

‘When I came back home, I felt pains, then I decided to sleep, then after some time, I woke up not feeling well at all and couldn’t sleep, when I tried to sleep my body was just somehow.’ (Participant 15, female, aged 32)

Sub-theme 1.2: Gastrointestinal system: The findings have shown that poor appetite and diarrhoea were experienced before treatment and continued even after taking the pills from the clinic. Participants expressed that these symptoms were present, and they would force themselves to eat because the tablets had been instructed to be taken with food. The findings of the study revealed that a single responder experienced diarrhoea after ingestion of the tablets from the clinic. The single participant had expressed that it was triggered by nausea. This symptom was described as not severe and occurred for a day and was expressed as the feeling of a thing that could not be released by the mouth and had to be released by the back. This was reported to occur on the day of treatment. As evidenced by the following quotes:

‘I was forcing myself to eat a bit because those pills needed me to take them with food.’ (Participant 17, female, aged 35)

‘But, after that I went to the clinic, and they gave me pills, I felt diarrhoea but not too much just one day, then my symptoms faded away, so the day I took the pills I did not vomit anything whatever that wanted to come out couldn’t come out through the mouth and had to come out by the back, but it just happened on that day only.’ (Participant 20, male, aged 54)

Sub-theme 1.3: Central nervous system: The findings revealed that certain symptoms experienced under this system included a pins-and-needles sensation, drowsiness, aphasia and confusion. These symptoms were mentioned to have been present continuously until they faded away. These symptoms were expressed to occur at any time of the day, when sitting or sleeping, and they were triggered by body pains. Aphasia was experienced by a single participant, who expressed that she could not speak, felt a fear of dying and could not understand what was happening to her body. It was expressed that the participant had complications while at home and was rushed to the hospital and admitted. It was reported that a single participant had experienced confusion, and it was described as a feeling of changes in the brain after completing the course of treatment.

This was evidenced by the following quotations:

‘I can’t explain it properly on what was happening on my legs, it was like they were not my legs the way they felt. The leg thing was still there, those symptoms faded away bit by bit until I felt my usual self, it was not easy at all. It was happening anytime, whenever I felt body pains, the feeling was there, even when I was seated, sleeping it was the same.’ (Participant 17, female, aged 35)

‘When I came back home, I felt drowsy and decided to sleep.’ (Participant 15, female, aged 32)

‘When I arrived home from the clinic I decided to sleep when I woke up, I was still not well at all, it nearly killed me. My body was just somehow, I could not even speak, they had to hire another car to take me to the hospital in Malamulele.’ (Participant 15, female, aged 32)

‘After I completed treatment, I felt like there was something wrong with my head, as if malaria had changed a few things in my head.’ (Participant 5, male, aged 46)

Sub-theme 1.4: Central and peripheral nervous system: It was reported that participants were unable to sleep at night even after taking treatment from the clinic. It was expressed that healing was gradual, and all symptoms faded away after the completion of the course of treatment. As evidenced by the following comment:

‘Hey hey hey, it took seven days still sleepless.’ (Participant 17, female, aged 35)

Sub-theme 1.5: Musculoskeletal system: The study’s findings also revealed that participants who experienced fatigue before treatment continuously experienced it after treatment from the clinic. Participants expressed that the feelings of self, including strength, returned gradually until full recovery. As evidenced by the following participant’s quote:

‘A whole week still weak, unable to do anything.’ (Participant 17, female, aged 35)

### Theme 2: Post-discharge experience from the hospital

The study’s findings revealed that a single symptom was experienced by a single participant. The results have also shown that patients who got treatment from the hospital had fewer symptoms than those coming from the clinic.

#### Symptoms on post-discharge from the hospital

Sub-theme 2.1: Post-discharge symptoms from the hospital: The study’s findings revealed that symptoms were still present even after discharge from the hospital. The symptoms were expressed to be less severe and participants healed gradually.

Sub-theme 2.2: Musculoskeletal system: The study’s findings revealed that participants experienced mild body weakness after discharge. Participants reported that this symptom was slight and gradually improved over time. While some participants mentioned that their recovery took longer than expected. Fortunately, all of them eventually healed completely and returned to their usual selves. As evidenced by the following quotes:

‘I was recovering bit by bit, felt tired.’ (Participant 16, male, aged 34)

‘I went to the clinic on the seventh day, the nurses gave me pills and transferred me to Malamulele hospital where they inserted a drip, I am not sure how long I stayed there, I got discharged but was still a bit weak for three months post-discharge.’ (Participant 9, male, aged 84)

‘I went home, and I was fine after three days post-discharge because I was still feeling weak and was scared of what had happened to me.’ (Participant 11, female, aged 24)

## Discussions

Participants reported persistent initial symptoms upon clinic arrival. This aligns with the WHO (2023), which stated that malaria is typically curable within 2 weeks with proper treatment; however, the finding suggests that symptoms persisted despite intervention. Furthermore, in contrast to the WHO’s (2023) observation that individuals usually feel better within days after successful treatment because of parasite clearance, the results indicate prolonged symptom experience. In addition, some participants developed new symptoms at home, different from their initial complaints. This concurs with the Centre for Disease Control and Prevention (2024), which found that infection-related symptoms can progress and persist even after treatment completion.

Participants reported having to eat despite poor appetite to take their prescribed medication, aligning with Nursing Revalidation’s (2024) emphasis on correct medication adherence to timing and dosage. This necessity explains their efforts to eat before taking tablets as instructed. The findings showed that initial symptoms such as fever, chills, body pain, weakness, and insomnia, continued after consultation but gradually improved towards recovery. Furthermore, some participants reported new symptoms, which included drowsiness, aphasia and confusion, experienced on the same day as their clinic visit. This observation aligns with Chelsea and William’s (2023) finding that drowsiness can be a malaria symptom, potentially linked to anaemia from parasite-induced red blood cell destruction.

Participants experienced aphasia; a finding consistent with Trivedi and Chakravarty’s (2022) report, which links aphasia to malaria as a neurological complication. This could explain the participant’s prolonged hospitalisation even after initial clinic treatment. The observed confusion also aligns with Fane’s (2021) case study of a patient with cerebral malaria who experienced confusion throughout hospitalisation, despite successful treatment for high parasitaemia. These observations are supported by studies indicating potential persistent symptoms post-clinic treatment.

Following hospital discharge, one participant reported a mild symptom. This aligns with Health Guide (2023), which found that mild fatigue can persist post-treatment as symptoms gradually subside. The participant experienced mild weakness upon discharge, less severe than during hospitalisation, with recovery progressing gradually. This is consistent with Health Guide’s (2023) information that fatigue and weakness can commonly last for several weeks after malaria treatment, and that the treatment itself can also cause weakness.

### Limitations

Some limitations have been identified in this research. The study was limited to Mhinga village of Vhembe district, Collins Chabane Municipality in Limpopo province. Similar research can provide different outcomes or conclusions if it is undertaken in a different place as findings cannot be extended to other villages and clinics. The objective of the study was to assess the post-discharge experiences of individuals diagnosed with malaria; however, the sample strategy encountered challenges because of incomplete, incorrect and absent personal information in the clinic record. Other participants could not be located and were unreachable by telephone for those reasons. This issue may have affected the results in some manner. Furthermore, a few participants were unable to share their complete experiences, as they had forgotten about them since they occurred a long time ago.

### Recommendations

Most households at Mhinga village are very clean, but there were a few that the researcher visited with unclean yards. During interviews, the participants would verbalise that they know the yard should be kept clean, but they are not practicing what they have been taught. Thus, there should be a dedicated supervision team, for instance, assigned by the chief, to ensure that control and prevention measures are maintained in households. Recommendations for research include investigating other villages regarding the experiences of people diagnosed with malaria to gather more information that might have been missed in this study.

## Conclusion

Following the discussion, the study concludes that the Mhinga community has experienced symptoms after discharge from the clinic and Malamulele Hospital, which were articulated to improve gradually until complete recovery and included both previously experienced symptoms and some new manifestations. The study objective was met following the research question that demands the post-discharge experiences of people diagnosed with malaria at Mhinga village in line with the interview guide that enquires the experiences of those diagnosed with malaria. Following the emerging main themes and subthemes, the post-discharge experiences of people diagnosed with malaria at Mhinga village were investigated, and the study’s research question was answered. Certain findings were consistent with earlier research conducted in various regions of the country.
